# How volatile components stabilize bulk nanobubbles: a model study

**DOI:** 10.3389/fchem.2026.1792068

**Published:** 2026-02-19

**Authors:** Jing Li, Zhenjiang Guo, Shuai Xia, Hongguang Zhang, Xianren Zhang

**Affiliations:** 1 College of Pharmaceutical Engineering, Jining Medical University, Jining, China; 2 State Key Laboratory of Organic−Inorganic Composites, Beijing University of Chemical Technology, Beijing, China; 3 Clinical Research Institute of Clinical Medicine, Nanjing Drum Tower Hospital, Medical School, Nanjing University, Nanjing, China

**Keywords:** bulk nanobubbles, dynamic equilibrium, molecular dynamics simulation, stability, volatile components

## Abstract

**Introduction:**

Although bulk nanobubbles have been widely studied, the contribution of volatile components to their stability remains largely unexplored.

**Methods:**

This work investigates the stabilizing effect of volatile components (e.g., CO2, short-chain hydrocarbons) on nanobubbles in liquid bulk. A thermodynamic model is established to account for the dynamic equilibrium between non-condensable gas (exemplified by oxygen) and volatile species inside the bubble. The model was developed and validated using molecular dynamics simulations.

**Results:**

The findings demonstrate that volatile components introduce a negative-feedback mechanism that balances gas exchange across the bubble interface, thereby enabling long-term stability. By contrast, systems containing only non-condensable gases fail to sustain stable nanobubbles.

**Discussion:**

This study reveals that the synergistic interaction between volatile and non-condensable gases is a key mechanism for stabilizing bulk nanobubbles.

## 1 Introduction

The extraordinary stability of interfacial nanobubbles (Lohse and Zhang, 2015a) has been reported and explained through several distinct mechanisms. These can be broadly categorized into interface-related mechanisms and bulk solution conditions. The former includes the contamination model (Ducker, 2009), the contact line pinning mechanism (Guo et al., 2016; Liu and Zhang, 2013; Weijs and Lohse, 2013; Zhang et al., 2013), and the high gas density model near the substrate (Peng et al., 2013). The latter encompasses conditions such as gas oversaturation (Lohse and Zhang, 2015a; Liu and Zhang, 2014). Of particular relevance to this work is the dynamic equilibrium model (Brenner and Lohse, 2008; Chen et al., 2015; Seddon et al., 2011), which attributes stability to a balance between gas influx and outflux, facilitated by a gaseous layer at the hydrophobic substrate-water interface (Brenner and Lohse, 2008). This model provides a foundational framework for considering gas exchange dynamics. However, it primarily addresses interfacial nanobubbles where a three-phase contact line is present.

In contrast, numerous studies have reported the observation of nanobubbles in bulk liquids (Chen et al., 2025; Li et al., 2016; Ohgaki et al., 2010; Zhang and Seddon, 2016; Zhu et al., 2016), demonstrating that bulk nanobubbles can also remain stable in the absence of triple-phase contact line pinning. The clear distinction between these two types of nanobubbles suggests that the stability of bulk nanobubbles cannot be fully explained by models developed for interfacial nanobubbles. In previous studies, several models have been proposed to explain the stability of bulk nanobubbles, including the hydrate shell model (Ohgaki et al., 2010), the insoluble gas model (Yarom and Marmur, 2015), models based on nanoparticle-shelled or amphiphile-coated interfaces (Li et al., 2025; Yasui et al., 2016), the interface charge model (Tan et al., 2020; Zhang et al., 2020), and the thermal fluctuation model (Chen et al., 2024; Wang et al., 2022). Among these, the hydrate shell model attributes the rigidity of bulk nanobubble surfaces to the formation of a hydrate layer. The insoluble gas model posits that nanobubble interiors contain gas with minimal water solubility. The nanoparticle-coated model proposes that adsorbed hydrophobic nanoparticles induce a dynamic gas flux equilibrium, analogous to interfacial nanobubbles (Brenner and Lohse, 2008). Amphiphilic molecules can reduce the interfacial tension at the bubble interface. Additionally, the charge model suggests that electrostatic interactions balance Laplace pressure, whereas the thermal fluctuation model holds that thermally driven capillary waves lower effective surface tension.

In the theoretical treatment of macrobubbles, volatile components are generally neglected. Their large curvature radii result in negligible Laplace pressure, keeping the internal pressure near ambient levels. Consequently, the behavior of volatile species is governed solely by their equilibrium vapor pressure, which is insensitive to minor volume changes. In contrast, for nanobubbles (<100 nm), high Laplace pressure (>5 MPa) markedly elevates internal pressure. While water vapor pressure remains low (∼3.17 kPa) and negligible, volatile species with relatively high critical pressures, such as CO_2_ (7.38 MPa), are driven toward phase-transition thresholds under such nanoscale confinement, significantly increasing their effective density within the bubble. Thus, the effect of volatile components in nanobubbles cannot be neglected during shrinking or growing processes, impacting their stability in bulk solution.

Aqueous solutions under ambient conditions often contain various trace volatile components (VCs), such as CO_2_, short-chain hydrocarbons, NH_3_, fluorocarbons and alcohol components. These VCs can be introduced into water through atmospheric dissolution, biological activity, and mineral interactions. When present within nanobubbles, these volatile components can influence bubble stability through two primary mechanisms. First, they can alter the bubble’s surface tension. Second, they may interact with the non-condensable gases (e.g., nitrogen and oxygen) inside the bubble. Both effects are crucial for determining the overall stability of nanobubbles.

In this work, we try to illustrate the effect of such volatile components in stability of bulk nanobubbles. Given the diversity and uncertainty of these volatile components, here we developed a simplified theoretical model to investigate how such volatile components interact synergistically with non-condensable gases within bubbles (using oxygen as an example) to influence the stability of nanobubbles. Finally, we also confirmed our theoretical explanation with molecular dynamics (MD) simulations.

## 2 Thermodynamic analysis

In a gas solution, inside a bubble there are two gaseous components: non-condensable gas and volatile components. The mass of the nanobubble can be written as

$$m_{total}=m_{v}+m_{g}.$$ (1)

The rate of the mass exchange across the bubble is thus given as

$$\frac{dm_{total}}{dt}=\frac{dm_{v}}{dt}+\frac{dm_{g}}{dt}.$$ (2)

with both the contribution from the gas molecules and that from volatile components inside the bubble. The former can be calculated with the equation given by Epstein and Plesset (Epstein and Plesset, 1950) for the bubble having a radius of R. Here, the diffusion-limited gas transport described by the Epstein–Plesset equation is applied as a first-order approximation, consistent with its extended use in modeling gas dynamics at the nanoscale (Lohse and Zhang, 2015b; Tan et al., 2020; Zhang and Lohse, 2023). Meanwhile, the contribution from volatile components is treated separately through a phase-change flux model, capturing their distinct kinetic behavior at the nanoscale interface.

$$\frac{dm_{g}}{dt}=4\pi R^{2}\kappa \left(c_{\infty }-c\left(R\right)\right)\left[\frac{1}{R}+\frac{1}{\sqrt{\pi \kappa t}}\right],$$ (3)

in which κ is the diffusion coefficient of gas molecules, $c_{\infty }$ and $c\left(R\right)$ are the gas concentration far away from and around the bubble, respectively. The gas concentration is given by Henry’s law corrected for Laplace pressure, as commonly applied in nanobubble thermodynamics (Epstein and Plesset, 1950; Lohse and Zhang, 2015a; Zhang and Lohse, 2023),

$$c\left(R\right)=\left(1+\frac{2\sigma }{RP_{0}}\right)c_{s}.$$ (4)

with $p_{0}$ the ambient pressure and $c_{s}$ the gas solubility.

The contribution from the volatile component can be calculated as Plesset and Prosperetti (1977),

$$\frac{dm_{v}}{dt}=4\pi R^{2}J,$$ (5)

in which the net mass flux J at the bubble interface is written as

$$J=\alpha {\left(\frac{R_{g}T}{2\pi M}\right)}^{1/2}\left(\rho_{v}^{e}-\rho_{v}\right).$$ (6)

Here α is the accommodation coefficient for evaporation, $R_{g}$ is the gas constant, $\rho_{v}$ is actual vapor density, and M is the molecular weight of the volatile component. The saturated vapor density $\rho_{v}^{e}$ in the bubble can be given by Dalton’s law,

$$\rho_{v}^{e}=\left(1+\frac{2\sigma }{P_{0}R}\right)x\rho_{v}^{0},$$ (7)

where x is the fraction of the molar volume of vapor in the bubble and $\rho_{v}^{0}$ is the vapor density under the atmospheric pressure. The total mass in the left of Equation 1 can be written as

$$m_{total}=m_{v}+m_{g}=\frac{4}{3}\pi R^{3}\rho_{v}+\frac{4}{3}\pi R^{3}\rho_{g}$$ (8)

In the present model, the vapor density ( $\rho_{v}$ ) and gas density ( $\rho_{g}$ ) in the bubble take the following forms: $\rho_{v}=\rho_{v}^{0}\left(1+\frac{2\sigma }{P_{0}R}\right)x$ and $\rho_{g}=\rho_{g}^{0}\left(1+\frac{2\sigma }{P_{0}R}\right)\left(1-x\right)$ . With Equations 3–8, and the change rate of bubble radius in Equation 2 can be written as

$$\frac{dR}{dt}=\frac{\alpha {\left(\frac{R_{g}T}{2\pi M}\right)}^{1/2}\left(\left(1+\frac{2\sigma }{P_{0}R}\right)x\rho_{v}^{0}-\rho_{v}\right)+\kappa \left(c_{\infty }-\left(1+\frac{2\sigma }{RP_{0}}\right)c_{s}\right)\left[\frac{1}{R}+\frac{1}{\sqrt{\pi \kappa t}}\right]}{\rho_{v}^{0}\left(1+\frac{4\sigma }{3RP_{0}}\right)x+\rho_{g}^{0}\left(1+\frac{4\sigma }{3RP_{0}}\right)\left(1-x\right)}.$$ (9)

**FIGURE 1:**
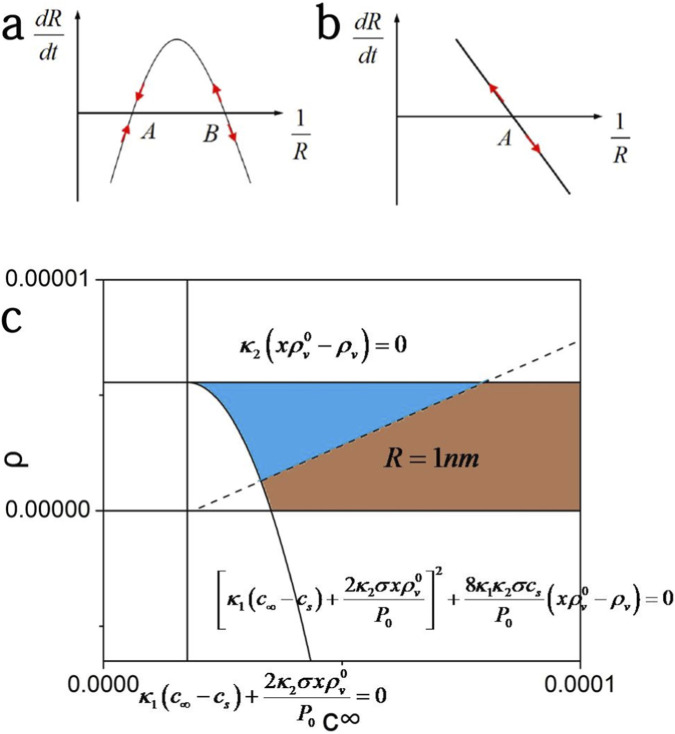
**(a)** Schematic representation of the stable (point A) and unstable (point B) equilibrium states in the phase space of dR/dt versus 1/R , as described by Equation 10. **(b)** Sketch of the unstable equilibrium point in the same phase space, corresponding to Equation 14. **(c)** Phase diagram illustrating the region of stable nanobubble existence (blue region) with radii larger than 1 nm in an oxygen-supersaturated aqueous solution.

In Equation 9, $\frac{1}{R}\gg \frac{1}{\sqrt{\pi \kappa t}}$ (Plesset and Prosperetti, 1977), so we can neglect the latter. We can solve the equation $\frac{dR}{dt}=0$ to obtain the state in dynamic equilibrium with its surrounding, the results indicate that

$$-\frac{2\kappa_{1}\sigma c_{s}}{P_{0}}{\left(\frac{1}{R}\right)}^{2}+\left[\kappa_{1}\left(c_{\infty }-c_{s}\right)+\frac{2\kappa_{2}\sigma x\rho_{v}^{0}}{P_{0}}\right]\cdot \frac{1}{R}+\kappa_{2}\left(x\rho_{v}^{0}-\rho_{v}\right)=0,$$ (10)

where $\kappa_{1}=\kappa$ and $\kappa_{2}=\alpha {\left(\frac{R_{g}T}{2\pi M}\right)}^{1/2}$ . Equation 10 is a quadratic equation of $\frac{1}{R}$ and thus it has two equilibria radii at most. However, one of them may not be a stable solution because the slight increase (decrease) in R must result in further decrease (increase) the mass inside the bubble, in other words, $\frac{dR}{dt}<0$ ( $\frac{dR}{dt}>0$ ). As a result, there is a negative feedback mechanism in keeping the bubble radius and which thus stabilizes the bubble both dynamically and thermodynamically. As shown in Figure 1a we can find schematically that point A is the stable one while B is not. The meaningful solution of Equation 10 (as point A) should meet the conditions as

$$\left\{\begin{array}{l} \kappa_{1}\left(c_{\infty }-c_{s}\right)+\frac{2\kappa_{2}\sigma x\rho_{v}^{0}}{P_{0}}>0\\ \kappa_{2}\left(x\rho_{v}^{0}-\rho_{v}\right)<0\\ {\left[\kappa_{1}\left(c_{\infty }-c_{s}\right)+\frac{2\kappa_{2}\sigma x\rho_{v}^{0}}{P_{0}}\right]}^{2}+\frac{8\kappa_{1}\kappa_{2}\sigma c_{s}}{P_{0}}\left(x\rho_{v}^{0}-\rho_{v}\right)>0 \end{array}\right.$$ (11)

and the stable equilibrium radius of bubble is obtained as Equation 12

$$R=\frac{4\kappa_{1}\sigma c_{s}}{P_{0}\left[\kappa_{1}\left(c_{\infty }-c_{s}\right)+\frac{2\kappa_{2}\sigma x\rho_{v}^{0}}{P_{0}}-\sqrt{{\left[\kappa_{1}\left(c_{\infty }-c_{s}\right)+\frac{2\kappa_{2}\sigma x\rho_{v}^{0}}{P_{0}}\right]}^{2}+\frac{8\kappa_{1}\kappa_{2}\sigma c_{s}}{P_{0}}\left(x\rho_{v}^{0}-\rho_{v}\right)}\right]}.$$ (12)

However, for non-condensable gas with much higher solubility, the effect of the volatile component becomes small enough so that it can be neglected, and there are no stable nanobubbles in the liquid as predicted by EP equation (Epstein and Plesset, 1950).

We solved Equation 11 for oxygen supersaturated solution at room temperature (298.15 K) and atmospheric pressure (101.325 kPa). Under the combined assumptions of Raoult’s law (constant vapor pressure), Henry’s law (constant dissolved gas equilibrium), and Dalton’s law, the vapor mole fraction x in the bubble remains independent of radius (Zhang et al., 2024). Therefore, the mole fraction of the volatile component was treated as a fixed value, and here it was taken as 0.011 as an example. The diffusion coefficient of oxygen was set to $3.49\times 10^{-5}\;cm^{2}/s$ (Krieger et al., 1967) and the saturated solubility of it is set to $8.9\times 10^{-6}\;g/cm^{3}$ (Zhang and Seddon, 2016). Surface tension is set to $0.071\times 10^{-2}\;N/\text{cm}$ . For vapor in the bubble, when gas constant $R_{g}$ is set as $8.314\times 10^{7}\text{erg}\cdot K^{-1}\cdot \text{mo}l^{-1}$ , the molecular weight M is assumed to 18.02 (Kieffer, 1977), α is set as one and the density of vapor under atmospheric is $0.5\times 10^{-3}\;g/cm^{-3}$ . Thus, the value of $\kappa_{2}$ can be solved to 3833.2. Finally, after solving Equation 11 we can obtain the phase diagram of the stable bubble as in Figure 1c, which shows the region of stable bubble with a radius larger than 1 nm.

However, what is the result when there are two or more types of gas molecules in the liquid while neglecting the effect of volatile components? We assumed that in the liquid there are two types of non-condensable gases with high solubility, so Equation 2 becomes Equation 13

$$\frac{dm_{total}}{dt}=\frac{dm_{g,1}}{dt}+\frac{dm_{g,2}}{dt}.$$ (13)

Both of the contributions of different gas molecules in the right of the equation were calculated by the EP equation. After simplifying and solving $\frac{dR}{dt}=0$ , the equilibrium radius of the bubble was obtained as

$$R=\frac{P_{0}}{2\sigma }\frac{\kappa_{1}\left(c_{\infty ,1}-c_{s,1}\right)+\kappa_{2}\left(c_{\infty ,2}-c_{s,2}\right)}{\kappa_{1}c_{s,1}+\kappa_{2}c_{s,2}}.$$ (14)

The equilibrium point was shown in Figure 1b, and there are no stable nanobubbles found since the negative feedback mechanism disappears. It is consistent with the experiments that most nanobubbles were observed to be filled with oxygen, nitrogen and hydrogen (Li et al., 2016; Zhang and Seddon, 2016).

## 3 Results and discussion

**FIGURE 2:**
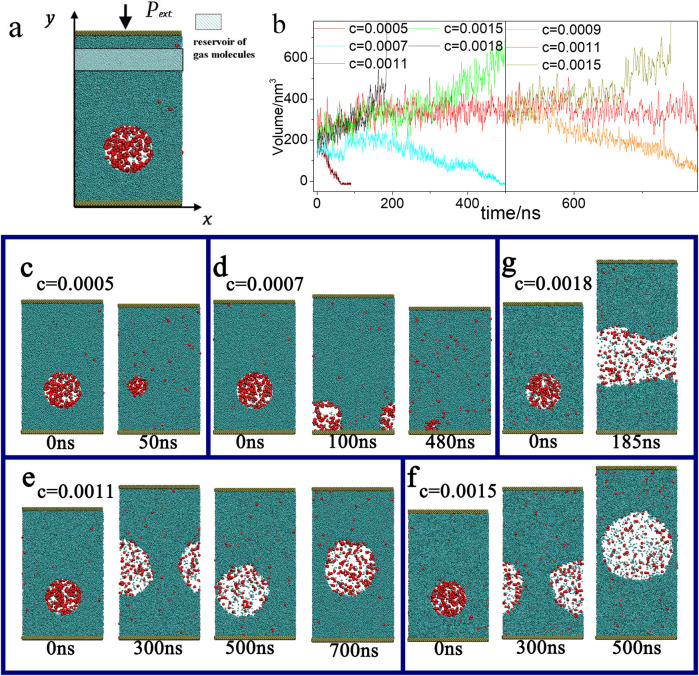
**(a)** Initial configuration of the MD simulation system. **(b)** Volume evolution of the nanobubble under different gas concentrations over simulation time. The right figure illustrates the change in nanobubble volume as a function of reservoir gas concentration, using the configuration at 500 ns with a gas concentration of 0.0011 as the initial state. **(c–g)** depict the temporal evolution of the nanobubble volume at different gas concentrations.

In this study, molecular dynamics (MD) simulations were performed using LAMMPS to investigate the coupling effect between volatile components and non-condensable gases. A quasi-two dimensional simulation box with dimensions of 22.4 × 2.24 × *H* nm^3^ was employed, as shown in Figure 2a, where *H* denotes the variable box height under given pressure. Periodic boundary conditions were applied in the x - and y -directions, while two confining flat substrates were placed at the top and bottom of the box along the z -direction. To simulate gas-liquid mixtures, a molecular reservoir was introduced to achieve the target gas concentration. Identity exchanges between liquid and gas molecules within the reservoir were carried out every 0.1 ns to maintain this concentration consistently. In the simulation, we model the volatile component as the solvent itself in its vapor phase. This is implemented by dynamically tagging solvent molecules when they evaporate into the nanobubble. This approach captures the essential phase-change physics while significantly simplifying the setup.

All intermolecular interactions were modeled using the Lennard-Jones (LJ) 12–6 potential, with parameter values listed in Table 1. Simulations were conducted in the isothermal-isostress (*NP*_*zz*_ *T*) ensemble, where pressure was controlled by applying an external force on the smooth top substrate while the bottom substrate remained fixed. The pressure and temperature were set to 3 atm and 99.2 K, respectively, resulting in a slight undersaturation of the pure liquid (Liu and Zhang, 2014). Under these conditions, the equilibrium solubility (mole fraction) of the non-condensable gas in the liquid is approximately 0.0004. The equations of motion were integrated using the velocity Verlet algorithm with a time step of 5 fs The system was first equilibrated for 10 ns Production runs were then performed for 500–1000 ns to track nanobubble evolution.

**TABLE 1:** Parameters for interaction between different molecules.

Interaction type	σ (nm)	ε (meV)	Cutoff (nm)
Liquid-liquid (LL)	0.34	10.30	1.1
Liquid-gas (LG)	0.34	3.26	1.1
Liquid-solid (LS)	0.34	6.87	1.1
Gas-gas (GG)	0.34	1.03	1.1
Gas-solid (GS)	0.34	1.89	1.1
Solid-solid (SS)	0	0	0

To create a nanobubble, 90% of the particles were removed from a circular region, and the initial gas-to-liquid ratio inside the bubble was set to 3:2. The bubble’s initial radius was set to 5.1 nm. Different gas concentrations were applied in the reservoir to observe the resulting volume change of the nanobubble, as summarized in Figure 2b. At a concentration of 0.0011, the nanobubble remained stable for over 750 ns (Figure 2e). Due to Brownian motion, the bubble moved upward and touched the source region after 750 ns, causing it to vanish abruptly within 1 ns. It should be noted that this disappearance does not imply inherent instability; rather, we posit that in the absence of such accidental contact, the nanobubble could remain stable for significantly longer periods.

When the gas concentration was lower than 0.0011 (e.g., c=0.0007 ), the nanobubble shrank rapidly and ultimately vanished (Figure 2c). In the case of *c* = 0.0007, the bubble first attached to the bottom substrate, showed a slight volume increase, and then progressively shrank until disappearance (Figure 2d).e 2d). For the intermediate value of 0.0015, the nanobubbles exhibit a slow growth trend and remain stable (Figure 2f). Conversely, at concentrations higher than 0.0015 (e.g., *c* = 0.0018), the nanobubble grew continuously, eventually undergoing a liquid-to-vapor transition (Figure 2g). In order to rule out potential influences from the initial bubble configuration, we used the stable nanobubble obtained after 500 ns in the simulation with c=0.0011 as the starting point for further simulations. The same trends were reproduced, as illustrated in Figure 2b.

**FIGURE 3:**
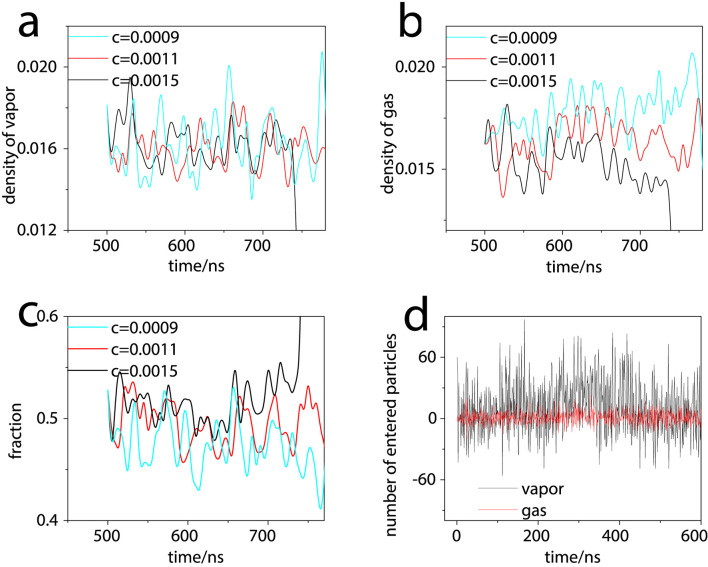
**(a–c)** Reduced density profiles within the nanobubble for different gas concentrations in the liquid: **(a)** vapor density, **(b)** gas density, and **(c)** vapor fraction. **(d)** Net flux of vapor and gas across the interface of a stable nanobubble at a gas concentration of 0.0011.

To elucidate the stability mechanism of bulk nanobubbles, we analyzed the reduced densities of the volatile component and non-condensable gas within the bubble over the time interval from 500 ns to 780 ns (Figure 3). The data reveal intense molecular exchange between the volatile component and the surrounding liquid phase. Specifically, the strong fluctuations in the volatile component effectively counteract the gas flux across the nanobubble interface, thereby inhibiting changes in bubble radius. As shown in Figure 3a, the vapor density remains approximately constant regardless of variations in bubble volume. In contrast, the density of the non-condensable gas increases with decreasing bubble size and decreases with increasing bubble size (Figure 3b), a behavior attributable to its lower diffusivity and interfacial content. This inverse relationship further corroborates the existence of a synergistic interaction between the volatile and non-condensable components during bubble volume changes. Consequently, the actual vapor density fraction within the bubble does not remain constant as predicted by an ideal model but varies with bubble volume (Figure 3c), indicating a dynamic coupling between the two gaseous phases.

The net fluxes of the volatile component and gas molecules across the interface of the stable nanobubble (c = 0.0011) are presented in Figure 3d, where positive values denote influx and negative values represent outflux. Both fluxes oscillate near zero, with the volatile component exhibiting significantly larger fluctuations due to its higher diffusivity. This behavior demonstrates a mutual compensatory effect between the volatile component and the non-condensable gas, which collectively stabilize the nanobubble—a finding consistent with our proposed theoretical model.

To further test this mechanism, we conducted simulations with two types of non-condensable gases present at high concentrations in the liquid. Under a wide range of conditions, no stable nanobubble was observed, consistent with the theoretical expectation that the absence of a volatile component disrupts the necessary dynamic equilibrium.

## 4 Conclusion

In conclusion, the volatile component acts as a rapid-response regulator, facilitating vigorous molecular exchange across the interface, while the non-condensable gas, governed by Laplace pressure and slower diffusion kinetics, responds more gradually to changes in bubble size. The near-zero net fluxes of both components reflect a dynamic balance: any tendency of the bubble to shrink or expand is offset by opposing mass transfers, creating a negative feedback mechanism that maintains the bubble at a stable equilibrium radius. These simulation results validate the thermodynamic predictions and confirm that the coexistence and interaction of volatile and non-condensable components are essential for the sustained stability of nanobubbles in bulk liquid.

In summary, we have proposed a possible mechanistic explanation for the stability of bulk nanobubbles, wherein a dynamic equilibrium arises from the interplay between non-condensable gases and volatile components within the bubble. This model not only provides a theoretical estimate for the stable nanobubble radius but is also corroborated by molecular dynamics simulations demonstrating long-term stability under suitable conditions.

## Data Availability

The original contributions presented in the study are included in the article/supplementary material, further inquiries can be directed to the corresponding authors.
